# Effects of SO_2_ Pollution on Household Insurance Purchasing in China: A Cross-Sectional Study

**DOI:** 10.3389/fpubh.2021.777943

**Published:** 2021-11-25

**Authors:** Ren Wang, Lizhi Zhang, Ting Tang, Fei Yan, Dawei Jiang

**Affiliations:** ^1^School of Finance, Hunan University of Technology and Business, Changsha, China; ^2^College of Science, Hunan University of Technology and Business, Changsha, China; ^3^Hunan Aerospace Hospital, Changsha, China

**Keywords:** SO_2_ pollution, commercial health insurance, residential health behavior, micro-data, discrete model

## Abstract

There have been considerable concerns regarding the effects of air pollution on health and economy over the past decades across the world. As insurance coverage has been closely related to household welfare, we aim to investigate the influence of air pollution, in particular, the sulfur dioxide (SO_2_) pollution on household purchases of commercial health insurance using data from the 2017 China Household Financial Survey (CHFS). The results show that the rise in SO_2_ emission has a significant positive association with tendency of residents to participate in commercial health insurance. The possibility of household commercial health insurance purchasing increases by 4% per 1,000 tons of SO_2_ emission. In addition, the proportion of commercial health insurance expenditure in household annual income increases by 29% per 1,000 tons of SO_2_ emission. The effects are also found to differ among resident groups. Residents in eastern parts of China are more likely to buy commercial health insurance facing SO_2_ pollution compared to those in western parts of China; people with higher income are more likely to be affected compared to those with lower income; families with the household head being female are more likely to be affected compared to those with the household head being male. This research provides baseline information on the formulation and implementation of future operation strategy in commercial health insurance companies of China.

## Introduction

Air pollution is one of the leading issues threatening human health. As the WHO suggested in 2016, almost 91% of people over the world lived in areas with major air pollutants higher than the WHO standard, and outdoor air pollution caused nearly 3.7 million deaths. In China, this problem is also concerning. Although the Chinese government has made persistent efforts on promoting air pollution control, like supporting the development of eco-industry park and green innovation (1, 2). Chinese residents were still exposed to fine particulate matter (PM_2.5_) three times higher than the standard stipulated in the WHO guideline. This situation mentioned above gives great significance in studying the health effect of air pollution. Existing research works provide numerous evidence that air pollution has a close association with the increasing morbidity of various diseases (3–6). Accompanied with rapid economic growth in the emerging economies is the severe ecological deterioration (7). In developing countries like China, the health effect of air pollution has attracted a lot of interest for their facing more serve environmental threats. China's efforts on reducing air pollutants emission is proved to be effective in lowering the premature death rates by 12.6% from 2013 to 2017 (8). Besides, air pollution is also found to have a negative effect on people's mental health in China (9). Sulfur dioxide (SO_2_) is one of the major air pollutants worldwide. Short-term exposure to SO_2_ is also proved to result in higher morbidity of cardiovascular diseases and increased hospitalization due to respiratory diseases including chronic obstructive pulmonary diseases and so on (10). It is also suggested that SO_2_ pollution notably correlates with a greater risk of acute myocardial infarction (11). The effect of SO_2_ pollution on the morbidity of diseases varies across the world due to differences in the concentration of the pollutant, lifestyles of residents, etc. In China, the morbidity of diabetes is found to increase by 3.835% with the concentration of SO_2_ raising every 10 μg/m (12). However, the risk of diabetes due to exposure to SO_2_ in the US is roughly 1.15 times that in China (13). In addition, exposure to SO_2_ also affects health expenditures of residents. According to the health investment model and the health capital depreciation theory developed by Grossman (14), the outside shock aroused by exposure to air pollutants including SO_2_ would accelerate the depreciation of health capital stock of residents. To maintain their health status, residents should expand their health expenditures. Therefore, many scholars have discussed the relationship between air pollution and medical cost of residents (15). Air pollution would significantly increase medical costs and healthcare costs of residents (16). Moreover, the effect of air pollution differs across regions in China. There exists a positive association between air pollution and medical expense in eastern and central regions, but a non-linear threshold effect is detected in the western region of China. Based on the micro-data of Chinese residents, it is found that fine PM_2.5_ has a positive association with medical care cost, hospitalization spending, and self-payed healthcare costs of residents (17). Furthermore, the study reveals that young people are more sensitive to the detrimental effect and the long-term effect is smaller. Other scholars study the effect in different regions over the world. In Iran, air pollution increases the healthcare cost either in the short run or in the long run (18). In Korea, the improvement in air quality is suggested to gain potential health benefits with the air quality index, which includes five major air pollutants being the indicator (19). A series of studies above confirm that air pollution would drive residents to increase their healthcare expenses. However, few researches focus on how to cope with this side effect that air pollution brought, which is of great necessity to improve people's well-being and promote public health.

For another, commercial health insurance is an important approach for residents to reduce their self-paid healthcare costs and hence relieve their economic burden of relevant diseases, which is helpful for improving household welfare. The earliest study on the effect of air pollution on the purchase and cancellation of commercial insurance contracts is conducted to analyze motives of consumers to purchase insurance and reveals a positive relationship, which is inconsistent with behavioral economics. Besides, evidence shows that the health and economic loss brought by air pollution can be reduced by taking avoidance response and medical insurance is found to be an effective measure (20). Health insurance could also effectively reduce the mortality rate, which therefore is of significance for residents to maintain fitness (21). Under the Patient Protection and Affordable Care Act, almost 90% of American citizens are covered by health insurance or something similar (22). Facing the debate over healthcare policies, Woolhandler et al. (21) summarize the evidence on the association between insurance coverage and mortality. The evidence shows that health insurance is helpful to reduce mortality.

In conclusion, considering that commercial health insurance could assist people in improving health conditions, it is meaningful to study the effects of SO_2_ pollution on household insurance purchasing, particularly for those developing countries that are still encountering serious environmental problems. Commercial health insurance purchasing could be an effective way to cope with the adverse health effects of air pollution on the local residents. Under the background of the “Healthy China Plan”, more efforts should be made to focus on public health, to provide health services of better quality, and to improve the existing public healthcare system. Therefore, this paper is intended to focus on the effects of SO_2_ pollution on household insurance purchasing from the micro-perspective and to provide necessary information on the benign operation of commercial health insurance companies of China. By matching the individual data from China Household Financial Survey (CHFS) with the provincial annual SO_2_ emission data in China, this study examines whether SO_2_ pollution would drive people to take avoidance actions, which increase their possibility and degree of commercial health insurance purchasing. Since insurance purchasing is a complicated process affected by many factors, to reach convincible results, besides common control variables like demographic characteristics, more specific individual factors are fixed including financial literacy, social trust, etc. Last but not least, considering the characteristics of our indicators and data, the Probit and Tobit models are adopted to capture the effects of SO_2_ pollution on household insurance purchasing.

## Materials and Methods

### Data

This paper focuses on the associations between levels of SO_2_ pollution and household commercial health insurance purchasing based on micro-data from the 2017 CHFS, which covers 400,011 interviewees from 29 provincial-level regions in China excluding Tibet, Xinjiang, Hong Kong, Macao, and Taiwan. The survey provides abundant data on household and individual characteristics including income and asset conditions, age, gender, risk attitudes, etc. Therefore, it provides necessary data for our empirical research considering that insurance purchase behavior is also affected by various household and individual factors. The data on SO_2_ pollution is from China Statistical Yearbook (2017). The pollution data are matched with micro-data through the provincial code listed in the dataset of the CHFS.

### Data Description

#### Dependent Variable

This paper analyzes the impact of SO_2_ pollution on household commercial health insurance purchasing from two aspects: the possibility of purchasing commercial health insurance and the degree of their purchase behavior. For defining household commercial health insurance purchasing, a household with at least one member covered by commercial health insurance is defined as a household covered by commercial health insurance. Therefore, we construct a dummy variable *Insurance* to indicate whether a household is covered by commercial health insurance, whose value is 1 when its members purchased commercial health insurance and is 0 when its members did not. For another, the degree of their purchase behavior is quantified by their proportion of commercial health insurance cost in household income. The data on the commercial health insurance cost is also obtained from the survey.

#### Independent Variable

The annual emission of SO_2_ pollutants is used as the core independent variable to measure the degree of SO_2_ pollution. The major air pollutants are including fine PM_2.5_, inhalable particles (PM_10_), SO_2_, nitrogen dioxide (NO_2_), carbon monoxide (CO), and ozone (O_3_). Some studies employ the Air Pollution Index (API) and Air Quality Index (AQI) released by the Ministry of Ecology and Environment of the People's Republic of China to quantify the air pollution in China. However, API is abolished in 2015 and AQI covers only 74 cities across China. In addition, the open average annual statistics on PM_2.5_, PM_10_, NO_2_, CO, and O_3_ are seldom collected. Considering that this paper is based on annual data, although the AQI, which is reported on a daily basis, could be more comprehensive, it would be difficult to be incorporated into our analysis. Therefore, this paper uses SO_2_ emission to quantify the degree of air pollution and focuses on the effect of SO_2_ pollution on the commercial health insurance purchase behavior of households.

#### Control Variable

To focus on the effect of SO_2_ pollution on household insurance purchasing as possible, it would be important to include a series of comprehensive control variables in the regression models. Insurance purchasing is a sophisticated process affected by many factors. People's health factors and other factors like financial literacy are necessary to be considered. According to the relevant studies, this paper includes various factors affecting commercial health insurance purchasing (23, 24). The control variables include two parts. First of all, the household characteristics are fixed including annual income, total assets, the proportion of medical expenditure in annual income, the proportion of children under 14 in the household, the proportion of the elder over 60 in the household, and residential background. The other part is the characteristics of the household head including age, gender, health conditions, marital status, education level, financial literacy, risk attitude, social health insurance, social trust, etc.

Several control variables are processed to eliminate potential data bias. First of all, the anomalous data with the values of annual income and total assets equal to or <0 is omitted. In addition, to eliminate the outliers, the indicators of annual income and total assets are winsorized at the upper and lower limit of 1% and then logarithmically processed. Second, based on the three questions about interest rate calculation, understanding of inflation rate, and knowledge of investment risks in the survey, this paper constructs three dummy variables, respectively, whose value is 1 if the answer is correct and is 0 if not. Those dummy variables are utilized to measure the financial literacy of the interviewee by employing factor analysis. All the variables are shown in Table 1.

**Table 1:** Variable definition.

**Variable name**	**Definition**
Insurance	Commercial health insurance, 1 for having commercial health insurance, 0 for not having
Cost	The proportion of commercial health insurance cost in household income
SDE	The annual SO_2_ emission
Income	The nature logarithm of household annual income
Residence	Residence background, 1 for rural household, 0 for urban household
Elder	The proportion of the elder over 60 in the number of household members
Children	The proportion of children under 14 in the number of household members
Medical	The proportion of medical expenditure in household annual income
Social	Social health insurance, 1 for having social health insurance, 0 for not having
Health	Self-rating health conditions, ranging from 1 to 5, 1 for the best, 5 for the worse
Risk	Risk attitude, ranging from 1 to 5, 1 for risk preference, 5 for risk aversion
Gender	Gender of the household head, 1 for male, 0 for female
AGE	Age of the household head
AGESQ	The square of age
Trust	Social trust, ranging from 1 to 5, 1 for the highest, 5 for the lowest
Education	Education level, 1 for a middle school education or below, 2 for high school education to junior college, 3 for undergraduate education or above
Financial	Financial literacy, quantified based on factor analysis of questions about the interest rate, inflation rate, and risk attitude in the survey

### Model Specification

Considering whether a household purchase commercial health insurance is a dummy variable, the Probit model is used to analyze the effect of SO_2_ pollution on the possibility of a household purchasing commercial health insurance. The estimation equation is shown as follow:


(1) $$\begin{array}{l} Insurance^{*}\;=\;\beta_{0}\;+\;\beta_{1}SDE\;+\;\alpha X\;+\;\varepsilon \\ \;Insurance\;=\;1(Insurance^{*}>0) \end{array}$$

The variable *Insurance* reflects whether a household has purchased commercial health insurance or not, whose value is 1 for households covered by commercial health insurance and is 0 for those not covered by commercial health insurance. The variable *SDE* is measured by SO_2_ emission volume. *X* represents all the control variables and ε is the error term.

Furthermore, this paper studies that to what degree SO_2_ pollution affects household commercial health insurance purchasing, which is measured by their proportion of commercial health insurance cost in household annual income. However, the commercial health insurance cost is zero for those households not purchasing commercial health insurance. That is to say, the variable *cost* is truncated. Therefore, this paper uses the Tobit model to analyze the association between SO_2_ pollution and the degree of household commercial health insurance purchasing. The estimation equation is listed as below:


(2) $$Cost\;=\;\beta_{0}\;+\;\beta_{1}SDE\;+\;\alpha X\;+\;\varepsilon$$

The variable *Cost* is the proportion of commercial health insurance cost in household annual income. The variables *SDE* and *X* are the same as those in equation (3).

### Empirical Analysis

#### Geographic Characteristics of SO_2_ Emission in China

**Figure 1:**
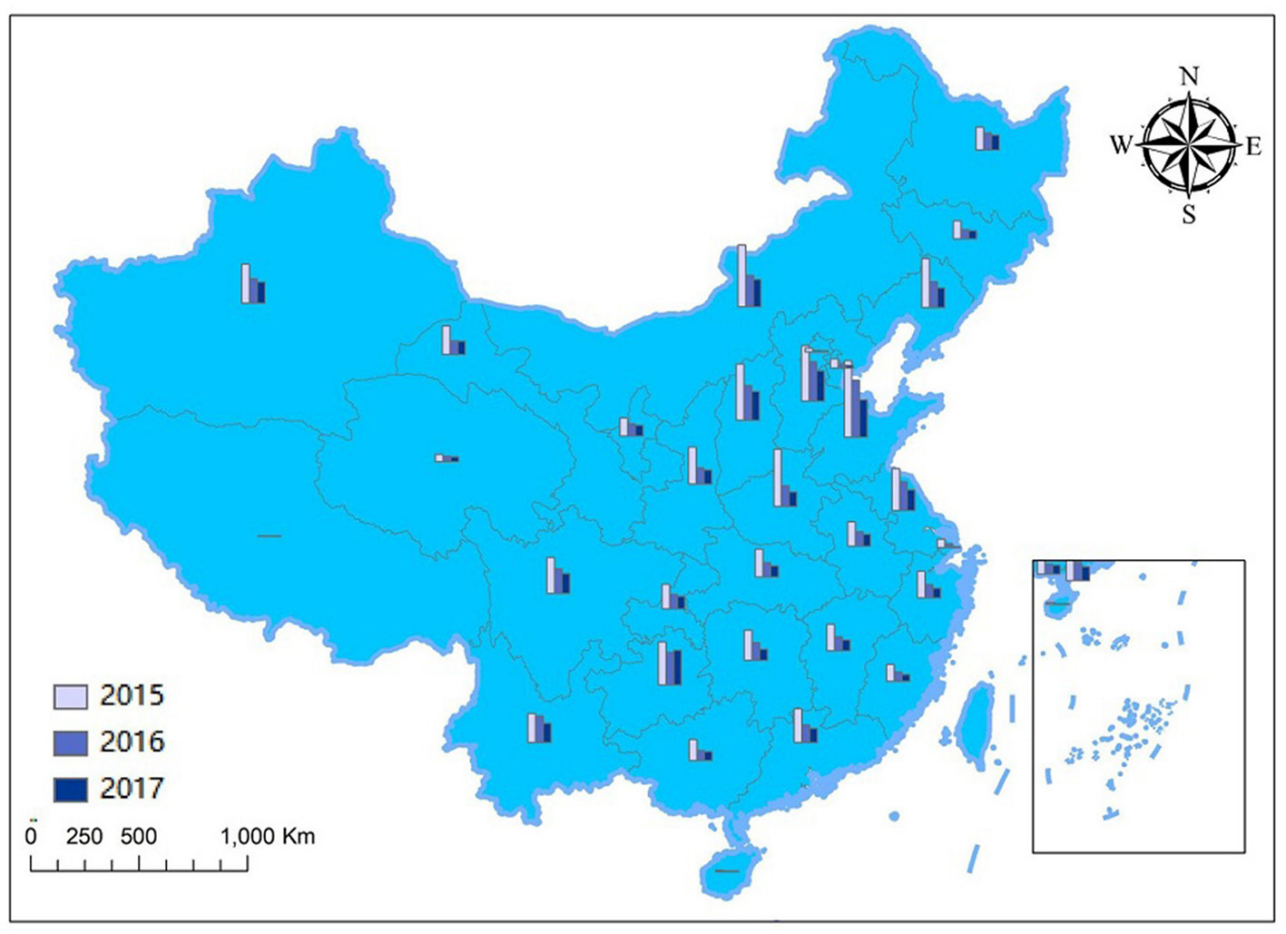
Changes in the SO_2_ emissions across provinces and regions from 2015 to 2017.

The changes in annual SO_2_ emissions of each province in China from 2015 to 2017 are shown in Figure 1 by ArcGIS. It is shown that annual SO_2_ emissions of every province appeared to decline from 2015 to 2017, which suggests that the overall air quality in China is improving. However, the annual SO_2_ emissions of several provinces were still at a high level, and the total annual SO_2_ emission in the eastern region was higher than that in the western region in 2017, which might be due to the disparity in the levels of economic development and industry agglomeration (25). In addition, improvement in reducing SO_2_ emission differed across provinces. The top three provinces in an effort to reduce SO_2_ emission were Henan, Shandong, and Inner Mongolia, which decreased annual SO_2_ emission by over 600,000 tons. The improvement was relatively nominal in the provinces where the annual SO_2_ emission was at a low level over the past few years like Tibet and Hainan. In general, from 2015 to 2017, the SO_2_ emission of provinces in China decreased significantly, which indicates that the Chinese government is committed to improving the atmosphere environment.

**Figure 2:**
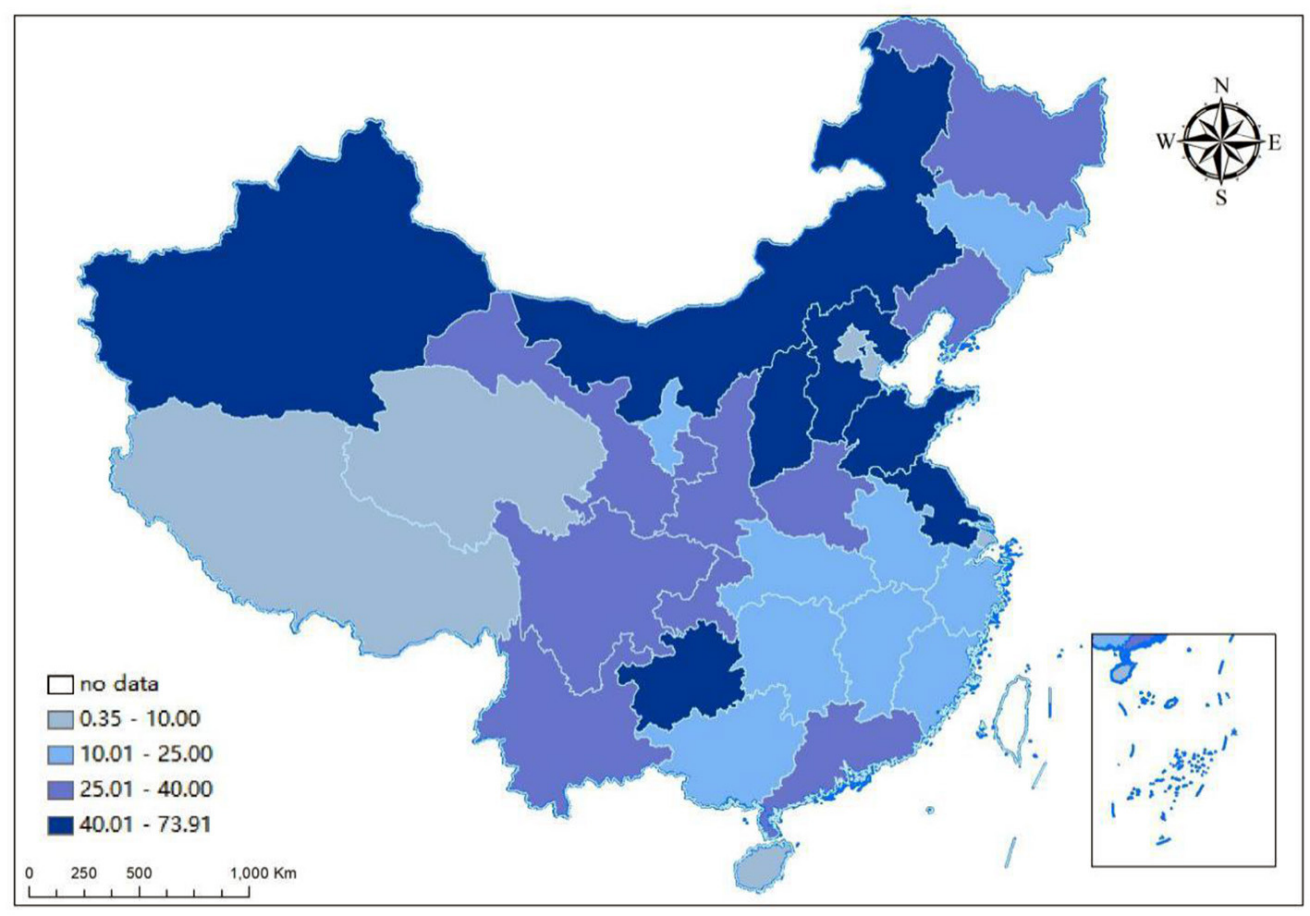
The geographic distribution of SO_2_ emissions across provinces and regions in 2017.

The geographic distribution of SO_2_ emissions over provinces in 2017 is shown in Figure 2. It shows that there observed notable spatial differences in annual SO_2_ emission across provinces. The highest annual SO_2_ emission amounted to 739,100 tons, whereas the lowest annual emission was only 3,500 tons. Particularly, the annual SO_2_ emissions of several provinces exceeded 400,000 tons, which might have posed a severe threat to the local atmosphere environment and the health of residents, including Shandong, Guizhou, Hebei, Shanxi, Inner Mongolia, and Xinjiang. In comparison, the annual SO_2_ emissions of some provinces and regions were at a lower level <100,000 tons like Beijing, Tianjin, Shanghai, Hainan, and Qinghai.

**Figure 3:**
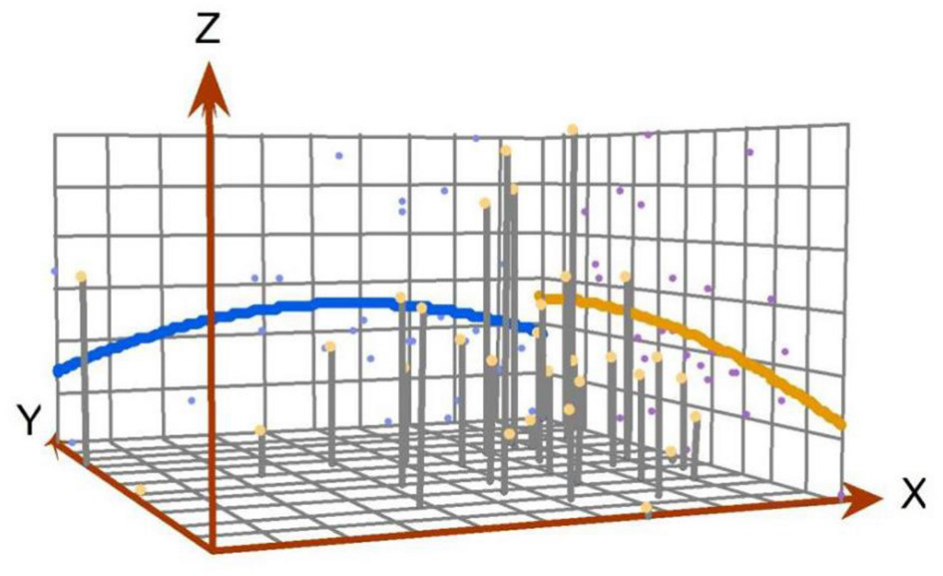
The perspective view of the geographic distribution of SO_2_ emissions in China.

Figure 3 is the perspective view of the geographic distribution of SO_2_ emissions over provinces and regions in 2017, which reflects the spatial distribution of the provincial SO_2_ emissions and their overall trend. The *X*-axis indicates the east–west direction in China, and the *Y*-axis indicates the north–south direction. The *Z*-axis measures the provincial annual SO_2_ emission. The fitted curves in the X–Z plane and Y–Z plane show the geographic distribution of provincial annual SO_2_ emission in the east–west direction and the north–south direction, respectively. Figure 3 shows that the fitted curve in the X–Z plane is inverted U-shaped and the fitted curve in the Y–Z plane is like an arc, which indicates that the 2017 provincial annual SO_2_ emissions in China were spatially uneven. Specifically, the annual SO_2_ emission in the central region of China was higher than that in the eastern and western regions, and the annual SO_2_ emission in the eastern region was relatively higher than that in the western region. As for the difference between the south and north, the annual SO_2_ emission in the northern part of China was higher than that in the southern part.

#### Summary Statistics

After omitting the missing value and outliers, 20,100 valid observations are acquired in total as the sample for the empirical analysis. The sample is divided into two groups by whether a household had purchased commercial health insurance or not. Table 2 shows the mean and SD for the main variables in the empirical analysis. It shows that the annual SO_2_ emissions are unevenly distributed across regions. The average proportion of households with commercial health insurance in the sample is 5.07%. Moreover, there exist apparent differences in household characteristics among the observations. The average annual income and total assets of households with commercial health insurance are higher than those of households without commercial health insurance. For those households without commercial health insurance, their average proportion of medical insurance in annual income is also higher than their counterpart, which suggests that medical costs could be a heavy economic burden for them to bear. As for the other variables, it is also shown that the average performance of households with commercial health insurance is better than that of households without commercial health insurance in education level, health conditions, social trust, risk attitude, and social trust.

**Table 2:** Summary statistics.

**Variable**	**With commercial health insurance**			**Without commercial health insurance**		
	**Observations**	**Mean**	**SD**	**Observations**	**Mean**	**SD**
SDE	19,081	29.875	18.300	1,019	29.850	18.371
COST	19,081	0	0	1,019	0.574	11.571
Income	19,081	10.541	1.472	1,019	11.486	1.211
Asset	19,081	12.515	1.863	1,019	13.716	1.420
Medical	19,081	76.143	8039.688	1,019	0.00004	0.0004
Elder	19,081	0.333	0.392	1,019	0.134	0.243
Children	19,081	0.078	0.134	1,019	0.139	0.155
Residence	19,081	0.376	0.484	1,019	0.190	0.393
AGE	19,081	56.965	13.382	1019	48.570	11.607
AGESQ	19,081	3424.099	1540.491	1,019	2493.646	1205.995
Gender	19,081	0.813	0.390	1,019	0.801	0.400
Marriage	19,081	0.868	0.338	1019	0.901	0.299
Education	19,081	1.372	0.596	1,019	1.769	0.751
Health	19,081	2.673	1.008	1,019	2.317	0.893
Social	19,081	0.945	0.227	1019	0.970	0.172
Trust	19,081	4.000	0.936	1,019	3.663	0.930
Financial	19,081	0.056	0.703	1,019	0.485	0.646
Risk	19,081	4.159	1.140	1,019	3.617	1.209

#### Results

The results of our empirical analysis are shown in Table 3. In order to reveal the effect of SO_2_ pollution on household commercial health insurance purchasing and its degree, this paper employs the Probit and Tobit models as mentioned above, which are denoted by model 1 and model 2, respectively. For the regression results using the Probit model, it is shown that there exists a significant association between SO_2_ pollution and household commercial health insurance purchasing with the value of coefficient being 0.004, which suggests that SO_2_ pollution would raise the possibility of a household purchasing commercial health insurance. As for the regression results using the Tobit model, the coefficient value of the *SDE* is 0.0029, which proves that SO_2_ pollution would drive a household with commercial health insurance to increase the investment in health insurance.

**Table 3:** Empirical results.

**Dependent variable**	**Probit**	**Tobit**
	**Model 1**	**Model 2**
SDE	0.004^***^ (0.001)	0.029^**^ (0.012)
Income	0.110^***^ (0.017)	0.001 (0.215)
Asset	0.095^***^ (0.014)	1.349^***^ (0.182)
Medical	−5.589 (12.685)	−351.389 (323.565)
Elder	−0.261^***^ (0.076)	−3.645^***^ (1.017)
Children	0.715^***^ (0.116)	7.015^***^ (1.535)
Residence	−0.061 (0.043)	−1.178^**^ (0.568)
AGE	0.025^**^ (0.010)	0.327^**^ (0.133)
AGESQ	−0.001^***^ (0.001)	−0.004^***^ (0.001)
Gender	−0.039 (0.043)	−0.365 (0.570)
Marriage	−0.113^*^ (0.058)	−1.042 (0.764)
Health	−0.017 (0.018)	−0.344 (0.243)
Education	0.080^***^ (0.027)	1.261^***^ (0.361)
Social	0.171^**^ (0.086)	2.579^**^ (1.169)
Trust	−0.064^***^ (0.018)	−0.873^***^ (0.238)
Financial	0.162^***^ (0.027)	1.689^***^ (0.360)
Risk	−0.047^***^ (0.014)	−0.608^***^ (0.186)
Cons	−4.309^***^ (0.349)	−42.615^***^ (4.726)
Observations	20,100	20,100
Pseudo R2	0.1310	0.0635

*The empirical analysis is conducted by Stata. Numbers in parentheses are standard errors*.

*** *p < 0.01*,

** *p < 0.05, and*

* *p < 0.1*.

The regression results of the control variables indicate that the annual income of a household and total asset have a significant effect on its commercial health insurance purchase behavior, and the proportion of expenditure on commercial health insurance in annual income expands as the scale of total asset enlarges. The coefficients of the proportion of medical expenditure are insignificant in both models, which may be attributed to the crowding-out effect of medical expenditure on commercial health insurance expenditure. The proportions of the elder and children in the number of household members are significant. However, the coefficient of *Elder* is negative, whereas the coefficient of children is positive. This indicates that demand for commercial health insurance might decrease as the situation of the aging population gets more severe in China, whereas the increase in the proportion of younger people might boost the demand. The coefficient of *Residence* is insignificant, which suggests that there exists no apparent distinction between the demand of urban residents and rural residents for commercial health insurance. This situation might occur because commercial health insurance has not been popularized in China. As for the variables of individual characteristics, the effects of *AGE, Education, Social, Trust, Financial*, and *Risk* are significant, whereas the effects of *Gende*r and *Health* are insignificant. Particularly, the effects of *AGE* on the possibility of a household purchasing commercial health insurance and its degree takes on inverted U-shaped, which means that the demand for commercial health insurance would increase at first and then decrease as the age of household head increases. The existence of adverse selection and moral hazard might exclude those people with a poor health condition from the commercial health insurance market, which could lead to the insignificant effect of health conditions on household commercial health insurance purchasing.

#### Heterogeneity Effects

It is mentioned that purchase decision of households on insurance is affected by various household and individual factors. Therefore, it is necessary to analyze its heterogeneous effects. This paper analyzes the heterogeneous effects of SO_2_ pollution on household commercial health insurance purchasing from four aspects including residential background, region, income level, and gender. All the results are shown in Table 4.

**Table 4:** Results of heterogeneity effect.

	**SO_**2**_ pollution**	**SD**	**Pseudo R2**	**Observation**
**A:**
Urban	0.003^**^	0.001	0.1276	12,738
Rural	0.004^***^	0.002	0.0847	7,362
**B:**
Eastern region	0.005^***^	0.001	0.1591	7,728
Central-western region	0.001	0.002	0.1103	12,372
**C:**
Upper income	0.004^***^	0.003	0.0972	10,050
Lower income	0.003^**^	0.004	0.0734	10,050
**D:**
Male	0.003^***^	0.001	0.1252	16,338
Female	0.006^***^	0.002	0.1634	3,762

*** *p < 0.01*,

** *p < 0.05*.

After dividing the sample into two groups by whether they are urban or rural residents, it is shown that the effect of SO_2_ pollution on household commercial health insurance purchasing is still significant for both groups. As for the effects on the residents in different regions, it suggests that there exists a positive association between SO_2_ pollution and household commercial health insurance purchasing in the eastern region and the central-northern region. However, the effect in the eastern region is still significant, whereas the effect in the central-northern region turns into insignificant. This indicates that the willingness of residents in eastern regions is stronger to address the outside shock of SO_2_ pollution by investing in commercial health insurance. After dividing the sample into two groups by the median annual household income, it is found that the effect is more notable for those with higher income. The households with higher annual income have more disposable income, so they are more likely to purchase commercial health insurance facing the SO_2_ pollution. Last but not least, considering gender differences, the result shows that women are more likely to depend on commercial health insurance to cope with the adverse effect of SO_2_ pollution compared to men.

#### Robustness Test

The effectiveness of our empirical results above might be mainly affected by two types of endogeneity including omitted variables and reverse causality. To address potential endogeneity, this paper employs the two-stage least squares (2SLS) method with an instrumental variable. A valid instrumental variable should satisfy two preconditions. First, the instrumental variable must be independent of the error term, which could be denoted by *cov*(*IV*, ε) = 0. In other words, the instrumental variable must be exogenous. Second, the instrumental variable must be highly related to the major dependent variable. According to a relevant study, this paper utilizes the annual precipitation amount at the provincial level as the instrument variable (26). For one thing, it is found that the precipitation could directly influence air pollutants like SO_2_ and hence is highly correlated to SO_2_ pollution (27–29). For another, considering that precipitation is a natural phenomenon, it is exogenous. Furthermore, precipitation is considered to have nothing to do with the health condition of residents, let alone their commercial health insurance behavior.

The regression results using an instrumental variable are shown in Table 5. The *F*-value in the first-stage least square regression is over 10, which indicates that the instrumental variable precipitation passes the Cragg–Donald test and is not a weak instrumental variable. In the 2SLS regression, the positive effect of SO_2_ pollution on commercial health insurance purchase behavior of households is also significant, and the value of its coefficient increases. It indicates that the effects of SO_2_ pollution on household purchases of commercial health insurance might be underestimated in our empirical analysis above due to endogeneity. The regression results with an instrumental variable are inconsistent with our empirical results, which suggests that our evidence on the positive association between SO_2_ pollution and households commercial health insurance purchasing is robust.

**Table 5:** Results of robustness test.

**Dependent variable**	**Endogeneity**		**Robustness**			
	**IV-Probit**	**IV-Tobit**	**IV-Probit**	**IV-Tobit**	**IV-Probit**	**IV-Tobit**
	**Model 1**	**Model 2**	**Model 3**	**Model 4**	**Model 5**	**Model 6**
SDE	0.010^***^ (0.002)	0.124^***^ (0.032)	0.021^*^ (0.005)	0.256^**^ (0.066)	0.010^***^ (0.003)	0.124^***^ (0.034)
Control	Fixed	Fixed	Fixed	Fixed	Fixed	Fixed
Observations	20,100	20,100	20,100	20,100	16,973	16,973
*F*-value	246.37	246.37	35.17	35.17	196.72	196.72
Wald test	8.74^***^	10.39^***^	15.18^***^	13.93^***^	6.45^**^	8.44^***^

*Numbers in parentheses are standard errors*.

*** *p < 0.01*,

** *p < 0.05, and*

* *p < 0.1*.

Considering that using the indicator of SO_2_ pollution only might result in measurement bias, To overcome potential selection bias, and test the robustness of our results the indicator of SO_2_ pollution is substituted with the annual emission of nitrogen oxide (NO_x_) to conduct a robustness test. The results are shown in Table 5 in the volumes of models 3 and 4. It shows that the coefficients of the effect are 0.021 and 0.256, respectively, which are both significant. The regression results after changing the indicator of air pollution shock also prove the robustness of our empirical results. In addition, considering that commercial health insurance purchase behavior of households is particularly affected by age, the regression results might be biased including those with the age of household head being too higher. Therefore, this paper conducts re-estimation based on the sample without observations with the age of household head over 70. The results are shown in Table 5 in the volumes of model 5 and model 6. After omitting parts of the observations, the results are still inconsistent with those in our empirical results above. In conclusion, the deepening of SO_2_ pollution has a significant positive association with the tendency of residents to participate in commercial health insurance, and the results are robust.

## Discussion

The air pollution aroused by nature and human activities poses a great threat to the health status of human beings, which leads to increased morbidity of various diseases including lung diseases, respiratory diseases, heart diseases, and specifically childhood diseases (30, 31). Although air pollution damages the health of people (32), investing in commercial health insurance might be an effective approach to fight against this kind of adverse effect. Therefore, the aggravation of air pollution might have associations with the possibility of a household purchasing commercial health insurance. Based on the micro-data from the 2017 CHFS, this paper studies the effects of SO_2_ pollution on household commercial health insurance purchasing with a perspective from exposome. Our empirical results prove that SO_2_ pollution has a significant positive effect on household commercial health insurance purchasing. The possibility of a household purchasing commercial health insurance and its expenditure on commercial health insurance increase as SO_2_ pollution gets worse.

There exist some research works on the effects of air pollution on medical cost (33, 34), but few study the association between air pollution and household demand for health insurance. Moreover, previous studies on this topic are conducted from a macro-perspective. To the best of our knowledge, Chang et al. (35) first study on the effect of air pollution on the demand for commercial insurance from a micro-perspective. Based on the transaction data from a large insurance company in China, they found that for each SD increase in the Air Quality Index, the sales of insurance contracts would rise by 7.2%. Their research is remarkable but still has some limitations. Compared to our study, their data are from a single company, which might lead to sample bias. In addition, they do not take the individual factors into consideration, which might lead to omitted variable bias. Therefore, this study includes a series of household and individual factors affecting the insurance purchase behavior of people based on the micro-data from the 2017 CHFS which makes it possible for us to better reveal the effects of SO_2_ pollution on households' health insurance purchasing.

Our empirical results show that there are positive associations between SO_2_ pollution and household commercial health insurance purchasing, which is inconsistent with the previous studies. According to a similar study on the effects of air pollution on the demand of people for insurance, it also finds that air pollution would significantly increase the possibility of a household buying insurance and its insurance expenditure, and the effect is more apparent for health insurance compared with life insurance (23). The mechanism of the effect of air pollution on household commercial health insurance purchasing could be explained from two perspectives. For one thing, the investment of consumers in commercial health insurance is in fact to fulfill their demand for health. People increase their investment in health capital and transfer their potential health risks through commercial health insurance. The influences of air pollution on the health conditions of people received great attention in the past two decades (36). And it is found that frequent exposure to air pollutants like SO_2_ is linked to higher morbidity of respiratory diseases (37). According to the Grossman model, the outside shock from air pollution would accelerate the depreciation of health capital stock of people, which would drive them to increase their investment in health capital to cover the loss. As a result, their demand for commercial health insurance would expand. For another, air pollution makes people feel a sense of anxiety and unsafe (38, 39). For Residents with long-term exposure to air pollution, their anxiety would last and accumulate. Under this circumstance, people would tend to seek insurance to cover their potential loss as Maslow's needs theory suggest that insurance meets the need for security of people.

The heterogeneous factors are also considered in this study. First of all, it is found that urban and rural residents react differently to SO_2_ pollution. Second, regional heterogeneity also exists. Third, the effect of SO_2_ pollution also varies among households at different income levels. Last but not least, the gender of the household head affects the effect of SO_2_ pollution as well. Previous studies also provide some evidence on the heterogeneous effects. Geruso (40) reveals that the demand for health insurance differs between younger and elder consumers, and between men and women considering their difference in the potential medical expenditure. Other scholars experiment on the effect of risk attitude on the behavior of people for selecting health insurance (41). They find that the demand for health insurance of people is also affected by their risk attitude, and people are more likely to transfer their potential risk through insurance as risk awareness increase. In summary, besides regional disparity in air pollution degree, more individual factors should be considered to gain reasonable regression results of the effect.

To our knowledge, there are few studies on the effects of air pollution on household commercial health insurance purchasing. This paper conducts an empirical analysis based on the data from a micro database, which enriches the relevant studies of the exposome field. Furthermore, considering the regional disparity in the level of air pollution (42), the demand for commercial health insurance differs across regions. Insurance companies should consider environmental factors when pricing their products. Therefore, commercial health insurance with a reasonable price could be popularized by market forces and then help to promote public health. For another reason, facing the differences in individual characteristics, insurance companies should also tailor their products to meet specific health needs of people.

There are also some limitations in this paper. First of all, since there is a lack of data on air pollution, this paper selects the annual emission of SO_2_ pollutants only being the indicator of air pollution and mainly focuses on the effect of SO_2_ pollution on the commercial health insurance purchase behavior of households. However, SO_2_ is just one of the major air pollutants and it cannot reveal the whole effect of air pollution. Second, for privacy consideration, CHFS does not open its data on the location information of interviewee under provincial level. Therefore, this study has to define SO_2_ pollution at the provincial level, which might encounter measurement bias. Future researches on this topic could be conducted through the following directions. More air pollutants and their spatial factors should be considered to reach comprehensive and robust findings. How to establish an effective commercial insurance mechanism balancing its for-profit nature and its ability to promote public welfare could also be a far-reaching direction for future works.

## Conclusion

In conclusion, based on the data from the 2017 CHFS, this paper employs a combination of the Probit and Tobit models to reveal the influence of air pollution, in particular, the SO_2_ pollution on household purchase of insurance. To address potential endogeneity issues, an instrumental variable of precipitation is utilized to conduct a 2SLS regression. Furthermore, considering insurance purchase behavior affected by multiple individual factors, we also perform a heterogeneity analysis to test the effects of SO_2_ pollution on different groups of residents. It is found that SO_2_ pollution has a significant positive effects on household commercial health insurance purchasing. Furthermore, the heterogeneity analysis shows that the effects differ across households with different characteristics including residential background, living regions, income level, and education level.

## Data Availability Statement

All data generated or analyzed during this study will be made available by the authors. Further inquiries can be directed to the corresponding author.

## Author Contributions

RW conceived of the presented idea and supervised the findings. LZ, TT, FY, and DW collected data and performed the computations. LZ and TT prepared the manuscript. All authors have made a substantial and intellectual contribution to this paper and approved it for publication.

## Funding

This research was funded by the National Social Science Foundation of China (Grant No. 19BJY161), Key Program Provincial Education Department of Hunan Province (Grant No. 20A122), and Natural Science Foundation of Hunan Province (Grant No. 2021JJ30197).

## Conflict of Interest

The authors declare that the research was conducted in the absence of any commercial or financial relationships that could be construed as a potential conflict of interest.

## Publisher's Note

All claims expressed in this article are solely those of the authors and do not necessarily represent those of their affiliated organizations, or those of the publisher, the editors and the reviewers. Any product that may be evaluated in this article, or claim that may be made by its manufacturer, is not guaranteed or endorsed by the publisher.
